# Comparative Assessment of Pulsed and Continuous LED UV-A Lighting for Disinfection of Contaminated Surfaces

**DOI:** 10.3390/life12111747

**Published:** 2022-10-31

**Authors:** Erik Kvam, Brian Davis, Kevin Benner

**Affiliations:** 1 GE Research, One Research Circle, K1 5D29, Niskayuna, NY 12309, USA; 2 Current, Greenville, SC 29607, USA

**Keywords:** UV-A radiation, 365 nm LED light, photoinactivation, disinfection, native photosensitizers

## Abstract

The germicidal efficacy of LED UV-A lighting has scarcely been compared in continuous and pulsed modes for contaminated surfaces. Herein, we compare the disinfection properties of pulsed versus continuous lighting at equal irradiances using a 365 nm LED device that replicates the doses of occupied-space continuous disinfection UV-A products. Representative organisms evaluated in this study included human-infectious enveloped and non-enveloped viruses (lentivirus and adeno-associated virus, respectively), a bacterial endospore (*Bacillus atrophaeus*), and a resilient gram-positive bacterium (*Enterococcus faecalis*). Nominal UV-A irradiances were tested at or below the UL standard limit for continuous human exposure (maximum irradiance of 10 W/m^2^). We observed photoinactivation properties that varied by organism type, with bacteria and enveloped virus being more susceptible to UV-A than non-enveloped virus and spores. Overall, we conclude that continuous-mode UV-A lighting is better suited for occupied-space disinfection than pulsing UV-A at equivalent low irradiances, and we draw comparisons to other studies in the literature.

## 1. Introduction

Typical hospital room cleaning can miss up to 50% of high-touch surfaces [1,2], and since environmental cleaning can reduce the risk of hospital-acquired infections (HAIs) [3,4], a continuous supplement to standard manual cleaning procedures is desired. Hospital room surfaces typically have less than 2-log_10_ bacteria per square centimeter, so even low levels of inactivation from supplemental technologies may reduce the risk of HAIs [5,6]. Prior studies of LED UV-A light (365 nm) have demonstrated effective surface decontamination of bacteria [7,8,9], including those associated with HAIs, using continuous low-dose irradiation. However, the photobiological community has scarcely compared the germicidal properties of LED UV-A light in pulsed- and continuous- modes. A handful of UV-A studies have compared pulsed exposures in the presence of environmental photosensitizers [10,11,12] (e.g., titanium dioxide or vitamin-rich media carryover), but to our knowledge no comparative data exists for microbes directly (i.e., via native photosensitizers). In contrast, germicidal studies exist for genotoxic UV-C light (200–280 nm) in pulsed and continuous modes [13,14,15,16], where it is generally understood that high-dose UV-C damages DNA and is not suitable for human-occupied spaces without safety controls [17,18].

Herein, we tested the disinfection properties of pulsed and continuous lighting at equal irradiances using a 365 nm LED device that replicates the nominal doses provided by occupied-space UV-A light disinfection products. Representative human-infectious viruses, spores, and bacteria were irradiated on dry surfaces to assess the relative germicidal efficacies of pulsed and continuous UV-A below the UL 8802 human safety limit (10 W/m^2^ × 8 h, or 28.8 J/cm^2^) for indoor lighting [17]. It is increasingly understood that germicidal mechanisms of UV-A light are distinct from genotoxic UV-C light and involve adventitious generation of oxidative free radicals from cellular photosensitizers including vitamin- and iron-based molecules [9,19]. While rapid disinfection is possible using UV-A light, the doses required to do so generally exceed safety limits for human exposure and thus face similar operational challenges as UV-C light. Consequently, occupied-space lighting products are intentionally operated within safe dose ranges to produce slower disinfection rates.

## 2. Materials and Methods

### 2.1. Microorganisms

*Enterococcus faecalis* were purchased from ATCC (catalog #47077). *Bacillus atrophaeus* endospores (derived from ATCC #9372) were purchased from Crosstex (catalog #VBA-104). Adeno-associated virus (AAV) particles encoding eGFP were purchased from GeneCopoeia (catalog #AA002) at ≥5 × 10^12^ genome copies/mL. Lentivirus particles encoding eGFP were also purchased from GeneCopoeia (catalog #LP303-100) at ≥10^8^ transduction units/mL.

### 2.2. Reagents

Brain-Heart Infusion (BHI) growth media for microbial culture was purchased from Teknova (catalog #B9993). Sterile water for injection (WFI) was purchased from Teknova (catalog #W6100) and used as a diluent for all microorganisms. Human HT1080 cells were purchased from Agilent (catalog #240109) and cultured in high-glucose Dulbecco’s Modified Eagle Medium (DMEM, Thermo Fisher Scientific, Waltham, MA, USA, catalog #11995065) containing 10% heat-inactivated fetal bovine serum (FBS) and 1× antibiotic-antimycotic. Polybrene was purchased from Millipore Sigma (catalog #TR-1003-G) to maximize the transduction efficiency of lentivirus using HT1080 cells.

### 2.3. LED Device

**Figure 1:**
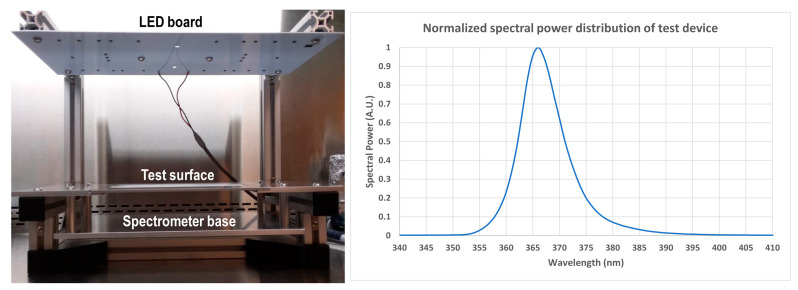
Test device (**left** panel) showing the LED UV-A board, the test surface where samples were irradiated, and the spectrometer base where the cosine corrector was placed to measure irradiance at the same plane as the test surface. The normalized spectral power distribution of this test device (**right** panel) has λmax at 366 nm and full width at half maximum of 9.1 nm.

The test fixture was configured to provide a time-averaged irradiance of 3 or 10 W/m^2^ of 365 nm UV-A in three different modes (constant irradiance, pulsed 10% duty cycle at 100 Hz, and pulsed 1% duty cycle at 100 Hz) at the test surface. These 100 Hz pulsed modes provide a higher instantaneous irradiance (~30 or 100 W/m^2^ for the 10% duty cycle or ~300 or 1000 W/m^2^ for the 1% duty cycle) in the form of repeated pulses of length 1 ms (10% duty cycle) or 100 µs (1% duty cycle). These pulses are delivered 100 times per second to produce a time-averaged irradiance of 3 or 10 W/m^2^, equal to the irradiance of the continuous modes. A 3 W/m^2^ irradiance replicates a typical irradiance from a UV-A disinfection lighting device on a surface of interest, as previously studied in situ for use in disinfecting medical equipment and patient rooms [7,8]. The 10 W/m^2^ irradiance represents the maximum permissible continuous human exposure permitted by UL standards for photobiological safety. Figure 1 depicts the test device and normalized spectral power distribution of the test fixture across both continuous and pulsed modes, while full spectrum data for each mode are summarized in Supplemental Figure S1 at the tested irradiance doses.

The device was constructed with a printed circuit board (PCB) populated with UV-A LEDs (NCSU276C, Nichia Corporation, Anan City, Tokushima Prefecture, Japan) located 200 mm from the test surface. The LEDs were arranged to maximize uniformity of irradiance over the 110 mm by 180 mm test surface. LED placement was determined by optical simulation (LightTools, Synopsys Inc., Mountain View, CA, USA) such that the average irradiance between test slides differed by less than 2%. The LEDs were driven by a programmable power supply (4400-10-56 LaserSource, Arroyo Instruments) capable of operating in constant-current and pulsed modes. Average irradiance was measured at the plane of the test surface using a UV spectrometer (Flame UV-VIS, Ocean Insight) equipped with a solarization-resistant fiber optic and right-angle cosine corrector. The spectrometer was calibrated for irradiance measurement by the manufacturer with the fiber and cosine corrector in place prior to the experiments. Irradiance measurements were taken as an average of 10 to 20 scans of 30 to 100 milliseconds each, to ensure that the length of the measurement was much longer than the cycle time for the pulsed configurations. Duty cycle and frequency through the LED PCB in operation were verified with a digital oscilloscope (TDS 3014C, Tektronix, Beaverton, OR, USA).

### 2.4. Experimental Design

For all germicidal tests, we intentionally eliminated potential sources of analytical bias during irradiation. For example, microorganisms were serially diluted in excess WFI (and irradiated in WFI) to avoid the artefactual effects of photosensitizing media components like vitamins [20,21,22]. Irradiated microbes were subsequently cultured using as few manipulations as possible to minimize shear after irradiation (in case microbes were rendered fragile but still viable). Finally, the LED UV-A light fixture was operated below the human exposure safety limit as defined by UL 8802, Outline of Investigation for UV Germicidal Equipment and Systems [17]. In the United States, UL 8802 is the industry-accepted standard for evaluating human exposure to UV from germicidal lighting. This standard applies the exposure limits and spectral weighting functions of IEC International Standard 62471 (Photobiological safety of lamps and lamp systems [23]) to occupied spaces. Exposure to UV-A is limited by the near-UV hazard exposure limit and the actinic UV hazard exposure limit. The near-UV (or UV-A) hazard exposure limit applies to 315–400 nm emissions and is 10 W/m^2^ unweighted irradiance for any exposure period greater than 1000 s and no greater than 8 h if continuously exposed (<28.8 J/cm^2^ cumulative dose). The actinic UV exposure limit is 30 J/m^2^ (spectrally weighted according to given functions) within any 8-h period. For the spectrum of the UV-A device used in this investigation, the weighting function is approximately 0.0001, which equates to 293 kJ/m^2^ unweighted dose, or 10.2 W/m^2^ irradiance for 8 h. Thus, the limiting factor is the near-UV hazard exposure limit of 10 W/m^2^ unweighted irradiance. For the repetitively pulsed 1% and 10% duty cycle modes used in this study, the exposure limits apply to the time-averaged irradiance values. Limits on UV-A and actinic exposures are also set by ACGIH in the form of Threshold Limit Values (TLVs^®^), which are identical to the IEC 62471 exposure limits [18]. Other standards or regulations may be used outside of the United States and may set different UV-A exposure limits. UV-A doses within the UL range have shown to be non-cytotoxic and non-irritating for fibroblasts and reconstructed epidermis [24,25].

#### 2.4.1. Bacteria and Endospore Irradiation and Recovery

*E. faecalis* was cultured on solid BHI from 10% glycerol stocks stored at −80 °C, and plates were disposed after 24 h to avoid the potential for spontaneous suppressor phenotypes. Colonies were inoculated in BHI broth and cultured for ~4 h to OD_600_ 1–2. Less than 10 μL of culture was then serially diluted in excess WFI to obtain an estimated density ≤ 2.3 × 10^5^ CFU/mL (to achieve ~3.5 × 10^2^ CFU after desiccation). Similarly, *B. atrophaeus* endospores (stock concentration ~2.5 × 10^5^ per mL in water) were also diluted in WFI to an estimated concentration of ~2.3 × 10^3^ spores/mL (to achieve ~3.5 × 10^2^ CFU after desiccation). These water inoculums (~0.9 mL) were transferred onto hydrophilic glass slides (Nunc Lab-Tek II CC2, Thermo Fisher, catalog #154739, wells removed prior to use) to improve bacterial viability during sample desiccation. Inoculums were dried overnight (~18 h) on these 8.6 cm^2^ surfaces under normal airflow within a Biosafety cabinet, and then dry surfaces were maintained in the dark or irradiated with UV-A light at a cumulative dose of ~4.3 J/cm^2^ (3 W/m^2^ × 4 h × 3600 s/h) continuously or at equivalent time-averaged irradiance using 1% or 10% duty cycle pulses. Dried slides containing *B. atrophaeus* endospores were also assayed at a higher cumulative dose of ~86.4 J/cm^2^ (10 W/m^2^ × 24 h × 3600 s/h) of LED UV-A light (Supplemental Figure S2). All slide samples were inverted onto solid BHI to “replica-plate” viable cells, analogous to contact plate assays for surface microbial testing. Inverted slides were pressed onto the agar using sterile tweezers, incubated for 2–5 min, and then removed prior to culturing the plate. Plate CFU growth was quantified using ImageQuant TL software 10.0.

#### 2.4.2. Adeno-Associated Virus Irradiation and Recovery

AAV particles were serially diluted in WFI to an estimated concentration of 4.63 × 10^10^ genome copies per mL. Approximately 0.45–0.9 mL of water inoculums (containing ~2.08 × 10^10^–4.17 × 10^10^ viral genomes) were transferred onto hydrophilic Nunc Lab-Tek II CC2 chambered slides (catalog #154852 or #154739, respectively) and then dried overnight (~18 h) on these 8 cm^2^–8.6 cm^2^ surfaces under normal airflow within a Biosafety cabinet. Dry surfaces were maintained in the dark or exposed to UV-A light at cumulative doses of either ~4.3 J/cm^2^ (3 W/m^2^ × 4 h × 3600 s/h) or ~14.4 J/cm^2^ (10 W/m^2^ × 4 h × 3600 s/h), which were applied continuously or under equivalent irradiances at 1% or 10% duty cycle pulses. After exposure, dry chambered slides were rehydrated with 2.7 × 10^5^–5.4 × 10^5^ HT1080 cells in 1–2 mL (respectively) of DMEM cell culture medium to titer AAV infectivity. HT1080 cells were cultured ≥48 h on the AAV-infected chamber slides, and infected GFP-positive cells were then quantified by flow cytometry.

#### 2.4.3. Lentivirus Irradiation and Recovery

Consistent with other groups [26], we found that lentivirus infectivity is severely impaired after surface drying (Supplemental Figure S3); consequently, our UV-A disinfection studies were conducted under aqueous conditions. Lentivirus particles were diluted in PBS (without calcium or magnesium) to an estimated concentration > 8.3 × 10^5^ transduction units per mL. Approximately 1 mL inoculums were aliquoted into 12-well culture plates (unlidded) and maintained in the dark or exposed to UV-A light at cumulative doses of either ~4.3 J/cm^2^ (3 W/m^2^ × 4 h × 3600 s/h) or ~14.4 J/cm^2^ (10 W/m^2^ × 4 h × 3600 s/h), which were applied continuously or under equivalent irradiances at 1% or 10% duty cycle pulses. After exposure, 0.3 mL sample aliquots (equivalent to ~2.5 × 10^5^ viral transduction units) were transferred into 24-well plates containing ~1 × 10^5^ HT1080 cells per well. Approximately 0.7 mL of transduction media was then added per well (DMEM containing 14.3% FBS and 5 ug/mL polybrene) and plates were cultured for ≥48 h. Infected GFP-positive cells were then quantified by flow cytometry.

#### 2.4.4. Flow Cytometry

Cells were recovered from culture wells and concentrated by centrifugation to roughly 1 × 10^6^ cells per mL. Cells were analyzed for GFP expression using the Cytoflex-S and CytExpert 2.4 software (Beckman Coulter). Viability and singlet gating using Forward Scatter and Side Scatter parameters were used to define subpopulations of individual viable cells. FITC gates for measuring the percentage of GFP expressing cells and mean fluorescence intensity were defined based on negative control cells lacking GFP expression. Serial dilution of input virus was performed to identify viral doses that provide predominantly single copy infections (Supplemental Figures S4 and S5), corresponding to ~20% total GFP-positive cells based on Poisson probability distributions. This approach facilitated comparison of mean fluorescence values, as cells infected with multiple copies of virus provide higher fluorescence intensity compared to single copy infections.

## 3. Results and Discussion

### 3.1. Continuous vs. Pulsed UV-A Irradiation of a Hardy Bacterium

**Figure 2:**
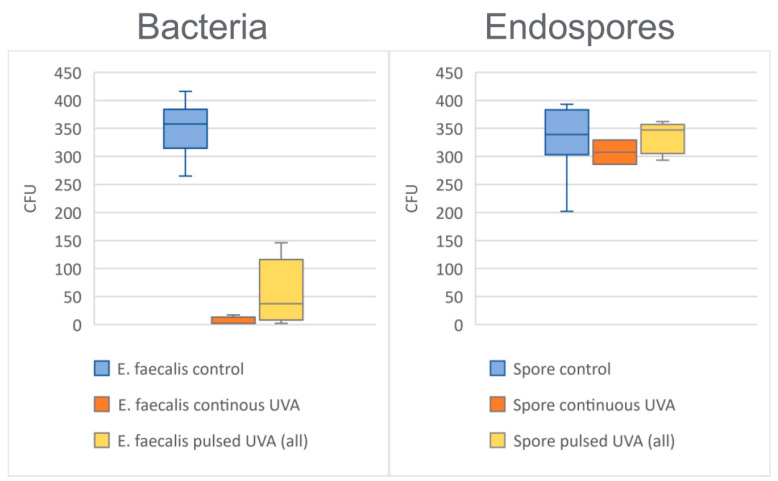
Surface viability of *E. faecalis* bacteria and *B. atrophaeus* endospores before and after low dose UV-A treatment (~4.3 J/cm^2^ either continuously or at equivalent time-averaged irradiance using 1% or 10% duty cycle pulses). Samples were prepared on 8.6 cm^2^ surfaces as described in Section 2.4.1 of the Experimental Design. Total colony-forming units (CFU) are enumerated and plotted across replicate samples (*n* ≥ 3). Independent results from 1% and 10% duty cycle pulses were pooled since both showed similar results and produced the same time-averaged irradiance (~4.3 J/cm^2^).

*Enterococcus faecalis* is a gram-positive bacterium notably resilient to challenging environments (such as desiccation) and therefore spreads easily in medical settings and contributes to hospital-acquired infection rates [27]. We compared the surface inactivation properties of UV-A light by drying *E. faecalis* onto slides to obtain ~40 CFU/cm^2^ (which is a realistic burden in hospital settings [6]) followed by irradiating at ~4.3 J/cm^2^ either continuously or using 1% or 10% duty cycle pulses. Contaminated slides were then inverted onto solid agar to “replica-plate” viable cells (analogous to standard contact plate assays for surface microbial testing). Figure 2 depicts the results of these experiments against dark-maintained controls. We observed ~98% inactivation (or 1.7 average log-reduction) upon continuous exposure of *E. faecalis*, which is consistent with our prior studies and translates to an average surface burden below 1 CFU/cm^2^ [9]. However, pulsed lighting at equivalent time-averaged irradiance achieved less potent inactivation at either duty cycle, resulting in ~80% average inactivation (or ~6 CFU/cm^2^ average surface burden) with considerably more variance than continuous lighting (Figure 2). These observations on dry surfaces agree with published studies by Mori [28] et al. who similarly concluded (in wet buffer environments) that continuous low-dose 365 nm light is more effective against bacteria than pulsing at similar low doses (i.e., below the 28.8 J/cm^2^ UL safety limit).

### 3.2. Continuous vs. Pulsed UV-A Irradiation of a Representative Endospore

*Bacillus atrophaeus* is a resilient bacterial endospore and is commonly used to qualify the sporicidal activity of biomedical decontamination methods. We tested the sporicidal activity of low-dose UV-A light by drying *B. atrophaeus* endospores onto slides to obtain ~40 CFU/cm^2^ and then irradiating at ~4.3 J/cm^2^ either continuously or using 1% or 10% duty cycle pulses. Figure 2 depicts the results of these experiments relative to dark-maintained controls after replica-plating viable cells from slide surfaces. We observed no significant inactivation of spores using low-dose UV-A in either continuous or pulsed modes (Figure 2) and extending the duration beyond the UL safety limit (to ~86.4 J/cm^2^ equivalent to 10 W/m^2^ × 24 h) failed to significantly improve the sporicidal performance of LED UV-A light (Supplemental Figure S2). These observations appear consistent with those published by Zhao et al. [29], who noted that dried spores require intense UV-A fluences (i.e., >50 W/m^2^) and/or supplementation with environmental photocatalysts to achieve robust surface inactivation. In contrast, non-spore forming bacteria are much more susceptible to UV-A photoinactivation, as illustrated by Figure 2 and in our previous studies [7,8,9].

### 3.3. Continuous vs. Pulsed UV-A Irradiation of a Representative Enveloped Virus

**Figure 3:**
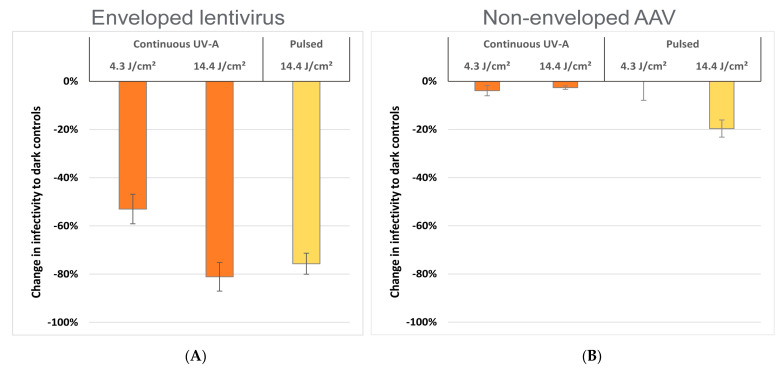
(**A**) Solution infectivity of enveloped lentivirus after low dose UV-A treatment (all data normalized to non-irradiated dark controls). Samples were prepared in saline buffer as described in Section 2.4.3 of the Experimental Design, and infected human cells were enumerated by flow cytometry across replicate samples (*n* = 4). Independent test results from 1% and 10% duty cycle pulses were pooled since both showed similar results and produced the same time-averaged irradiance (~14.4 J/cm^2^). (**B**) Surface infectivity of non-enveloped AAV after low dose UV-A treatment (data normalized to non-irradiated dark controls). Dried viral inoculums were prepared according to Section 2.4.2 of the Experimental Design, and AAV-infected human cells were enumerated by flow cytometry across replicate samples (*n* = 3). Independent test results from 1% and 10% duty cycle pulses were pooled since both showed similar results and produced the same time-averaged irradiance values (~4.3 J/cm^2^ or ~14.4 J/cm^2^).

Recombinant lentiviruses are derived from human immunodeficiency virus (HIV) and are used as delivery vehicles for clinical gene therapy. Lentiviruses are enshrouded by a lipid-containing membrane known as the viral envelope (similar to HIV and human coronaviruses); consequently, enveloped viruses are generally inactivated by drying alone. We found that lentivirus infectivity is severely impaired by surface desiccation (Supplemental Figure S3) consistent with other groups [26], so we utilized aqueous buffer preparations to assess the virucidal properties of UV-A light. Following lentivirus irradiation at 4.3 or 14.4 J/cm^2^, samples were transferred onto human tissue culture, and subsequent infection (i.e., expression of a lentiviral green fluorescent protein reporter gene) was analyzed by flow cytometry. Figure 3 summarizes lentiviral infectivity after normalizing to dark-maintained control virus. We observed that 14.4 J/cm^2^ UV-A significantly impaired the infectivity of lentivirus by almost 80% on average (or ~0.7 log reduction) compared to non-irradiated virus, with comparable efficacy between continuous and pulsed modes. Continuous irradiation at 4.3 J/cm^2^ produced less potent inactivation (~53% reduction, or ~0.3 log). These findings appear consistent with published observations by Rathnasinghe et al. [30] using 405 nm LED light, in which other lipid-enveloped viruses were significantly inactivated across similar dose ranges.

### 3.4. Continuous vs. Pulsed UV-A Irradiation of a Representative Non-Enveloped Virus

Adeno-associated viruses (AAV) are common human viruses not known to cause disease and are therefore used clinically in human gene therapy. AAV contains a protective protein-based shell called a capsid and lacks a lipid envelope, and therefore exemplifies a non-enveloped virus. Non-enveloped viruses are generally more tolerant of desiccation; consequently, AAV can be dried onto surfaces and maintain high infectivity unlike lipid-enveloped lentivirus [26]. We compared the virucidal properties of low-dose UV-A light by drying AAV onto slides and then irradiating at 4.3 or 14.4 J/cm^2^ continuously or using 1% or 10% duty cycle pulses. Treated slides were then seeded with cultured human cells, and AAV-infected cells (expressing an AAV green fluorescent protein reporter gene) were analyzed by flow cytometry. Figure 3 depicts the results of these experiments relative to dark-maintained controls. We observed that UV-A light was largely ineffective at reducing AAV surface infectivity, with the slight exception that pulsed irradiation at 14.4 J/cm^2^ decreased AAV infectivity by ~20% (or ~0.1 log reduction) compared to continuously-irradiated samples. When cross-compared to our lentiviral results in Figure 3, these AAV observations are consistent with published studies for 405 nm LED light, which similarly note that non-enveloped viruses are more resilient to photoinactivation than lipid-based enveloped viruses [30].

## 4. Conclusions

In total, our results demonstrate that LED UV-A light achieves low-level disinfection of vegetative (non-spore forming) bacteria and lipid-containing enveloped virus when applied at or below UL standards for human exposure. UV-A in pulsed modes proved less potent against vegetative bacteria compared to continuous UV-A (as shown by others [28]). For enveloped virus, comparable potency was observed between pulsed and continuous UV-A, but neither LED modes resulted in significant sporicidal activity. Only a slight benefit of pulsing UV-A at 14.4 J/cm^2^ was observed against non-enveloped virus. Overall, in neutral environments (i.e., where intrinsic microbial photosensitizers dominate), we conclude that continuous-mode UV-A lighting is better suited for supplemental disinfection than pulsing at equivalent low doses. Certain limitations exist in our study design; for instance, the potential influence of temperature or humidity were not exhaustively explored, and additional fomite materials were not the focus of our study. Nonetheless, we demonstrate that continuous-mode UV-A is a promising approach for microbial disinfection without additional pulsing cycles or exogenous catalysts. Further studies may extend upon these results with additional microbes, fomite materials, and environmental conditions.

The UV-A pulse frequencies tested in our study are within the range investigated by others (0.1–1000 Hz), and our results are consistent with Mori et al. [28] (who tested 10 Hz at 10% duty cycle) but discordant to Li et al. [12] (who reported optimal microbial inactivation with 100 Hz pulsed UV-A versus continuous mode). Since Mori et al. conducted experiments in neutral PBS buffer but Li et al. created biofilms from rich media, we believe that carryover of rich media (containing UV-A excitable vitamins and other potential photocatalysts) likely explains the published benefit of pulsed UV-A. Analogous studies have confirmed that media components such as vitamins artificially enhance the virucidal properties of blue LED light [22,31]. It is well known that environmental supplementation with photosensitizers and photocatalysts (e.g., photodynamic therapy) further augments the performance of LED UV-A light to achieve high-level disinfection and sterilization against spores and viruses [29,32].

While pulsed lighting can provide system-level benefits (such as power density, energy efficiency, or heat dissipation) relative to continuous lighting, our study demonstrates that pulsing provides no comparative biological advantage alone. By implication, environmentally-supplied photosensitizers or photocatalysts are likely required to achieve more favorable disinfection rates relative to continuous UV-A. This has important implications for real-world studies (e.g., soiled surfaces, shaded surfaces, etc.) where the contribution of environmental photosensitizers (including human secretions) is less understood but may naturally enhance the germicidal kinetics of disinfection lighting through indirect mechanisms [31,32,33].

## Data Availability

The data presented in this study are available on request from the corresponding author.

## Supplementary Materials

The following supporting information can be downloaded at: https://www.mdpi.com/article/10.3390/life12111747/s1, Figure S1: Time-averaged spectral irradiance characterization of the LED emission at the plane of the test surface; Figure S2: Surface viability of *B. atrophaeus* endospores before and after UV-A treatment exceeding the industry safety limit (~86.4 J/cm^2^, or 10W/m^2^ × 24 h duration); Figure S3: Representative lentiviral infection results before and after surface drying; Figure S4: Aqueous dilution of lentivirus to identify single-copy infections in HT1080 human cells; Figure S5: Dilution and drying of AAV for optimal infection in HT1080 human cells.

## References

[1] Xie A., Rock C., Hsu Y.J., Osei P., Andonian J., Scheeler V., Keller S.C., Cosgrove S.E., Gurses A.P. (2018). Improving daily patient room cleaning: An observational study using a human factors and systems engineering approach. IISE Trans. Occup. Ergon. Hum. Factors.

[2] Carling P.C., Parry M.M., Rupp M.E., Po J.L., Dick B., Von Beheren S. (2008). Improving cleaning of the environment surrounding patients in 36 acute care hospitals. Infect. Control Hosp. Epidemiol..

[3] Donskey C.J. (2013). Does improving surface cleaning and disinfection reduce health care-associated infections?. Am. J. Infect. Control.

[4] Han J.H., Sullivan N., Leas B.F., Pegues D.A., Kaczmarek J.L., Umscheid C.A. (2015). Cleaning Hospital Room Surfaces to Prevent Health Care-Associated Infections: A Technical Brief. Ann. Intern. Med..

[5] Weber D.J., Rutala W.A., Sickbert-Bennett E.E., Kanamori H., Anderson D. (2019). Continuous room decontamination technologies. Am. J. Infect. Control.

[6] Schmidt M.G., Attaway H.H., Sharpe P.A., John J., Sepkowitz K.A., Morgan A., Fairey S.E., Singh S., Steed L.L., Cantey J.R. (2012). Sustained reduction of microbial burden on common hospital surfaces through introduction of copper. J. Clin. Microbiol..

[7] Livingston S.H., Cadnum J.L., Benner K.J., Donskey C.J. (2020). Efficacy of an ultraviolet-A lighting system for continuous decontamination of health care-associated pathogens on surfaces. Am. J. Infect. Control.

[8] Brons J.A., Bierman A., White R., Benner K., Deng L., Rea M.S. (2020). An assessment of a hybrid lighting system that employs ultraviolet-A for mitigating healthcare-associated infections in a newborn intensive care unit. Light. Res. Technol..

[9] Kvam E., Benner K. (2020). Mechanistic insights into UV-A mediated bacterial disinfection via endogenous photosensitizers. J. Photochem. Photobiol. B Biol..

[10] Prasad A., Gänzle M., Roopesh M.S. (2019). Inactivation of Escherichia Coli and Salmonella Using 365 and 395 nm High Intensity Pulsed Light Emitting Diodes. Foods.

[11] Xiong P., Hu J. (2013). Inactivation/reactivation of antibiotic-resistant bacteria by a novel UVA/LED/TiO2 system. Water Res..

[12] Li J., Hirota K., Yumoto H., Matsuo T., Miyake Y., Ichikawa T. (2010). Enhanced germicidal effects of pulsed UV-LED irradiation on biofilms. J. Appl. Microbiol..

[13] Sholtes K., Linden K.G. (2019). Pulsed and continuous light UV LED: Microbial inactivation, electrical, and time efficiency. Water Res..

[14] Nyangaresi P.O., Qin Y., Chen G., Zhang B., Lu Y., Shen L. (2019). Comparison of the performance of pulsed and continuous UVC-LED irradiation in the inactivation of bacteria. Water Res..

[15] Song K., Taghipour F., Mohseni M. (2018). Microorganisms inactivation by continuous and pulsed irradiation of ultraviolet light-emitting diodes (UV-LEDs). Chem. Eng. J..

[16] Inagaki H., Saito A., Kaneko C., Sugiyama H., Okabayashi T., Fujimoto S. (2021). Rapid inactivation of SARS-CoV-2 variants by continuous and intermittent irradiation with a deep-ultraviolet light-emitting diode (DUV-LED) device. Pathogens.

[17] (2021). Outline of Investigation for UV Germicidal Equipment and Systems.

[18] Threshold Limit Values (TLVs®), Biological Exposure Indices (BEIs®) (2021). American Conference of Governmental Industrial Hygienists (ACGIH®). https://www.acgih.org/science/tlv-bei-guidelines/.

[19] Bosshard F., Bucheli M., Meur Y., Egli T. (2010). The respiratory chain is the cell’s Achilles’ heel during UVA inactivation in Escherichia coli. Microbiology.

[20] Stoien J.D., Wang R.J. (1974). Effect of near-ultraviolet and visible light on mammalian cells in culture II. Formation of toxic photoproducts in tissue culture medium by blacklight. Proc. Natl. Acad. Sci. USA.

[21] Tomb R.M., Maclean M., Herron P.R., Hoskisson P.A., MacGregor S.J., Anderson J.G. (2014). Inactivation of Streptomyces phage ϕC31 by 405 nm light: Requirement for exogenous photosensitizers?. Bacteriophage.

[22] Tomb R.M., Maclean M., Coia J.E., Graham E., McDonald M., Atreya C.D., MacGregor S.J., Anderson J.G. (2017). New Proof-of-Concept in Viral Inactivation: Virucidal Efficacy of 405 nm Light Against Feline Calicivirus as a Model for Norovirus Decontamination. Food Environ. Virol..

[23] IEC 62471. Photobiological Safety of Lamps and Lamp Systems. International Electrotechnical Commission (IEC) 2006. https://www.iecee.org/dyn/www/f?p=106:49:0::::FSP_STD_ID:7076.

[24] Besaratinia A., Kim S.-I., Bates S.E., Pfeifer G.P. (2007). Riboflavin activated by ultraviolet A1 irradiation induces oxidative DNA damage-mediated mutations inhibited by vitamin C. Proc. Natl. Acad. Sci. USA.

[25] Lelièvre D., Justine P., Christiaens F., Bonaventure N., Coutet J., Marrot L., Cotovio J. (2007). The episkin phototoxicity assay (EPA): Development of an in vitro tiered strategy using 17 reference chemicals to predict phototoxic potency. Toxicol. Vitr..

[26] Reuter J.D., Fang X., Ly C.S., Suter K.K., Gibbs D. (2012). Assessment of hazard risk associated with the intravenous use of viral vectors in rodents. Comp. Med..

[27] Fiore E., Van Tyne D., Gilmore M.S. (2019). Pathogenicity of Enterococci. Microbiol. Spectr..

[28] Mori M., Hamamoto A., Takahashi A., Nakano M., Wakikawa N., Tachibana S., Ikehara T., Nakaya Y., Akutagawa M., Kinouchi Y. (2007). Development of a new water sterilization device with a 365 nm UV-LED. Med. Biol. Eng. Comput..

[29] Zhao J., Krishna V., Hua B., Moudgil B., Koopman B. (2009). Effect of UVA irradiance on photocatalytic and UVA inactivation of Bacillus cereus spores. J. Photochem. Photobiol. B: Biol..

[30] Rathnasinghe R., Jangra S., Miorin L., Schotsaert M., Yahnke C., Garcίa-Sastre A. (2021). The virucidal effects of 405 nm visible light on SARS-CoV-2 and influenza A virus. Sci. Rep..

[31] Hessling M., Lau B., Vatter P. (2022). Review of Virus Inactivation by Visible Light. Photonics.

[32] Callahan S.M., Wonganan P., Obenauer-Kutner L.J., Sutjipto S., Dekker J.D., Croyle M.A. (2008). Controlled inactivation of recombinant viruses with vitamin B2. J. Virol. Methods.

[33] Terrosi C., Anichini G., Docquier J.D., Gori Savellini G., Gandolfo C., Pavone F.S., Cusi M.G. (2021). Efficient Inactivation of SARS-CoV-2 and Other RNA or DNA Viruses with Blue LED Light. Pathogens.

