# Supplementary material for: Comparative Assessment of Pulsed and Continuous LED UV-A Lighting for Disinfection of Contaminated Surfaces
Source: Life (Basel). 2022 Oct 31;12(11):1747. doi: 10.3390/life12111747 (PMC9696731; doi:10.3390/life12111747)
Supplement: Supplementary file 1 [file life-12-01747-s001.zip › life-1958429-supplementary.pdf]

# Comparative assessment of pulsed and continuous LED UV-A lighting for disinfection of contaminated surfaces

**Erik Kvam[1*], Brian Davis[1], and Kevin Benner[2]**

[1] GE Research, One Research Circle, Niskayuna, NY, 12309, USA

[2] Current, Greenville, SC, 29607 USA

**Supplemental Material**

Supplementary Figure S1 (pg. 2)

Supplementary Figure S2 (pg. 3)

Supplementary Figure S3 (pg. 4)

Supplementary Figure S4 (pg. 5)

Supplementary Figure S5 (pg. 6)

**Supplementary Figure S1.**

A B

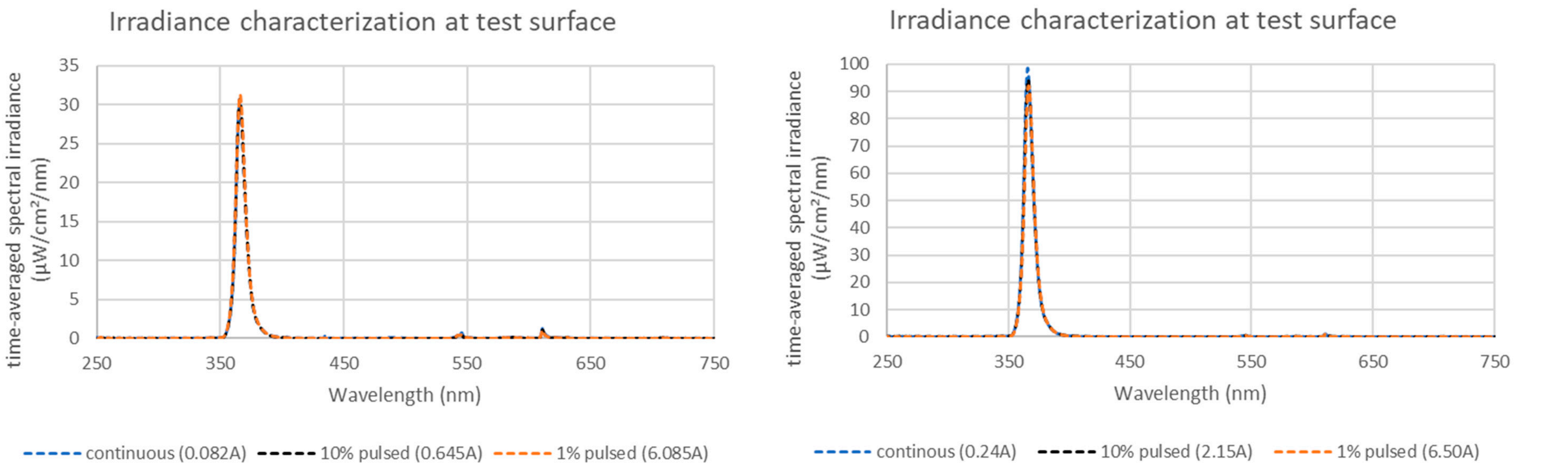


*Supplementary Figure S1*. Time-averaged spectral irradiance characterization of the LED emission at the plane of the test surface. Noted in parenthesis are peak LED drive currents to achieve UV-A time-averaged irradiances of ~3W/m$^2$ in Panel A or ~10W/m$^2$ in Panel B across continuous and pulsed modes. Average irradiance was measured using a UV spectrometer (Flame UV-VIS, Ocean Insight). Trace contaminate peaks are visible > 400nm due to overhead lights near the test environment.

**Supplementary Figure S2**.

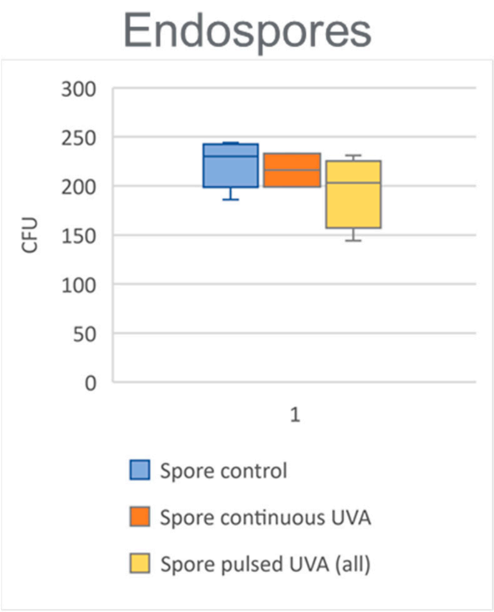


*Supplementary Figure S2*. Surface viability of *B. atrophaeus* endospores before and after UV-A treatment exceeding the industry safety limit (~86.4 J/cm$^2$, or 10W/m$^2$ x 24hrs duration). Samples were prepared and colony-forming units (CFU) were analyzed as described in Section 2.4.1 of the Experimental Design. No significant sporicidal activity is observed for continuous or pulsed UV-A exposures relative to dark controls.

**Supplementary Figure S3.**

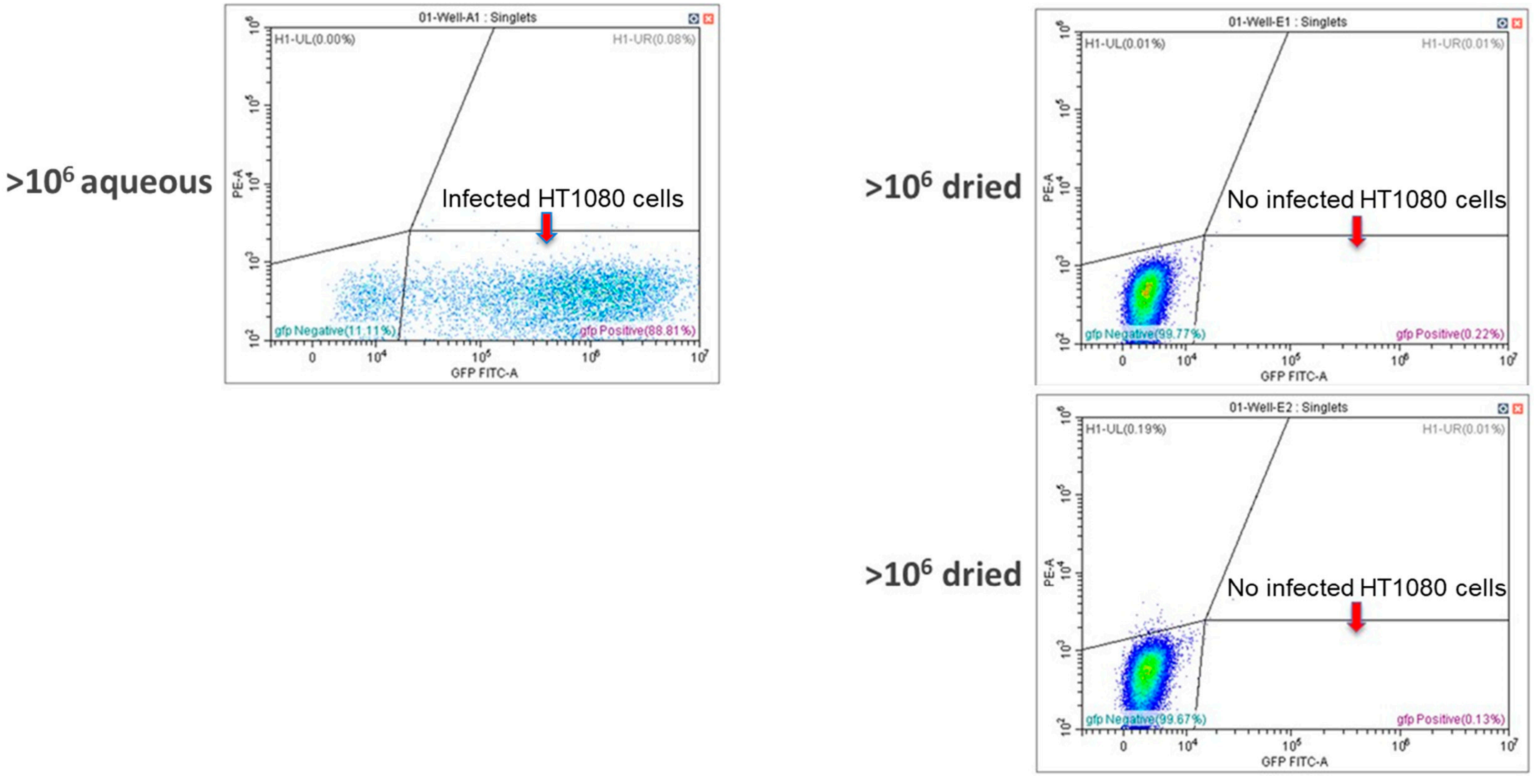


*Supplementary Figure S3*. Representative lentiviral infection results before and after surface drying. Water inoculums containing >$10^6$ transduction units of lentivirus were dried overnight on hydrophilic Nunc Lab-Tek II CC2 chambered slides, analogous to Section 2.4.2 methods for adeno-associated virus. Following culture with human HT1080 cells, no lentiviral-infected cells were observed by flow cytometry compared to liquid control virus. Infected cells express green fluorescent protein (GFP) as a reporter molecule.

**Supplementary Figure S4.**

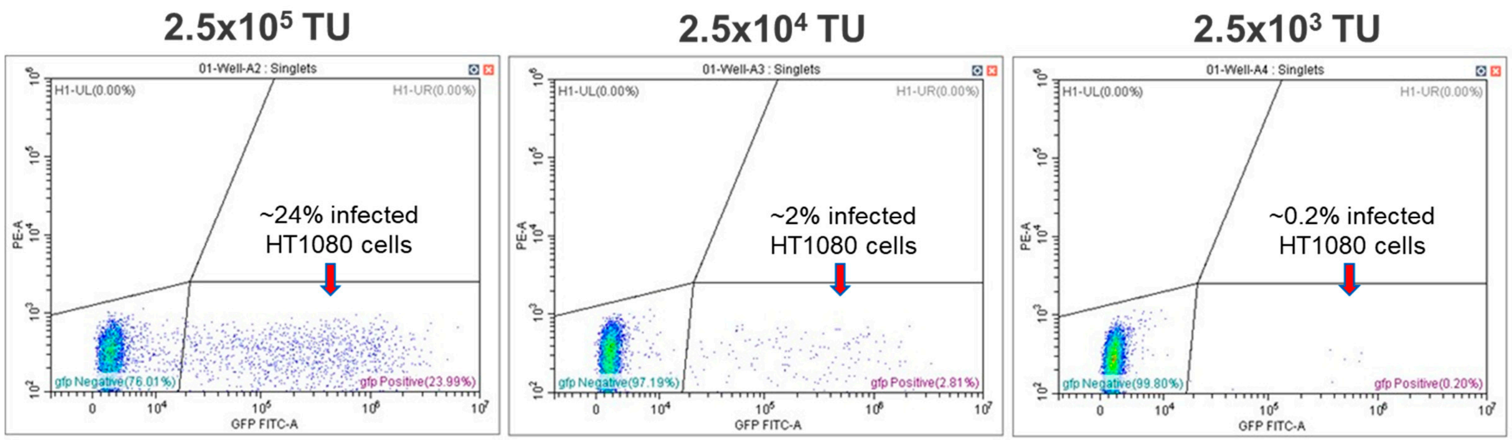


*Supplementary Figure S4*. Aqueous dilution of lentivirus to identify single-copy infections in HT1080 human cells. Lentivirus particles were serially diluted in PBS (without calcium or magnesium) to the concentration range listed above each panel. Based on these results, ~$2.5x10^5$ transduction units of lentivirus was down-selected as an optimal range to conduct UV-A exposure tests, since the assay sensitivity for HT1080 infection shows a linear response at 1-log and 2-log lower amounts of lentivirus.

**Supplementary Figure S5.**

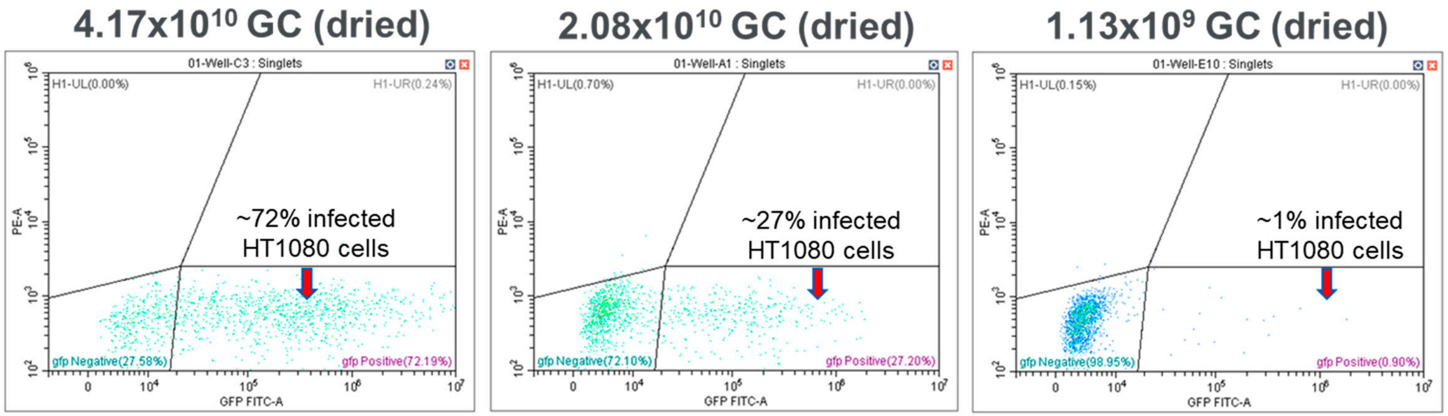


*Supplementary Figure S5*. Dilution and drying of AAV for optimal infection in HT1080 human cells. AAV particles were diluted in water to the concentration range listed above each panel, dried overnight on hydrophilic Nunc Lab-Tek II CC2 chambered slides, and then culture with human HT1080 cells as described in Section 2.4.2 of the Experimental Design. Based on these results, ~$2.08 \times 10^{10}$ – $4.17 \times 10^{10}$ genome copies (GC) of AAV were down-selected as an optimal range to conduct dry UV-A exposure tests since assay sensitivity for infection is at least 1-log lower.
